# Supplementary material for: Motives and Barriers Related to Physical Activity and Sport across Social Backgrounds: Implications for Health Promotion
Source: Int J Environ Res Public Health. 2021 May 28;18(11):5810. doi: 10.3390/ijerph18115810 (PMC8198157; doi:10.3390/ijerph18115810)
Supplement: Supplementary file 1 [file ijerph-18-05810-s001.zip › ijerph-1208420-supplementary.pdf]

Supplementary Table S1

Characteristics of included studies

Author(s), title, year	Country(-ies)	Study design and method	Population(s)	Aim and variable categories	Main results and conclusions
Alexandris, K. and B. Carroll. *Motives for recreational sport participation in Greece: Implications for planning and provision of sport services.* European Physical Education Review, 1997	Greece	Quantitative, cross-sectional, survey study	340 adults (51% females and 49% males) aged 18 – 65 years	To investigate the motives for taking part in recreational sports activities amongst adults and across sociodemographic variables	26 items were categorised into six motives: status, relaxation, intellectual, social, competition and health/fitness. The most important motive was recreational sports activities to expected health benefits, then the relaxation motive, followed by competition, intellectual, social. The least important motive was status.<br><br>There were differences in the motives across sociodemographic variables:<br>Singles scored higher than married persons in the status and competition motive.<br>The status motive decreased with age.<br>College students scored higher in the motives for the intellectual dimension, status dimension, competition dimension. University graduates scored lower in the social dimension as a motive for physical activity
Ball, K., et al. *How can socio-economic differences in physical activity among women be explained? A qualitative study.* Women and Health, 2006	Australia	Qualitative, cross-sectional, interview study	56 women aged 18-65 years (19 from a high-,<br>19 from a mid-, and 18 from a low-socio-economic status area	Investigate mediating factors that may explain differences in physical activity between women with varying Socio-economic status (SES)	The study identifies eleven themes: 1) activity type (high socio-economic status (SES) more likely to be involved in organised sports), 2) history of physical activity (parental participation and positive experiences with physical activity particularly prominent for high SES), 3) lack of time (especially pronounced for low/average SES who experienced lack of flexibility and fatigue as barriers), 4) planning (high SES more often had fixed routines for physical activity), 5) lack of motivation (especially low SES), 6) value of inactive activities (especially TV watching , most pronounced for low SES), 7) social constraints and support (high SES in particular felt guilty about prioritising physical activity over family and low and medium SES in particular cultivated physical activity alone), 8) work (low and medium SES in particular

					experienced work pressure and fatigue as a barrier), 9) safety and aesthetics in the local area (particularly a barrier for low SES), 10) access to facilities in the local area (not mentioned as a significant barrier), 11) expenses (not mentioned as a significant barrier)
Ball, K., et al. *Personal, social and environmental determinants of educational inequalities in walking: A multilevel study.* Journal of Epidemiology and Community Health, 2007	Australia	Quantitative, cross-sectional, survey study	1,282 women from different levels of socioeconomic disadvantage	Investigate mediating factors (personal, social, and environmental factors) that may explain differences in walking between women from different social classes. A distinction is made between walking for leisure and walking as transport	The study shows that a combination of personal, social, and environmental factors contributes to a lower level of leisure walking for women with a low education. The study mentions that further research is needed to better understand the pattern of how education may affect walking as transportation
Bird, S., et al. *Factors influencing the physical activity levels of older people from culturally-diverse communities: An Australian experience.* Ageing and Society, 2009	Australia	Quantitative, cross-sectional, mixed methods study	332 elderly persons aged 60 or more. Immigrants to Melbourne from Great Britain, Croatia, Greece, Italy, Macedonia, Malta, and Vietnam.	Investigate the barriers to physical activity for older people from different countries (cultures)	Personal barriers to physical activity, such as poor health, lack of energy, lack of motivation, and fear of getting hurt were widespread in all seven groups. People from the UK and Italy indicated fewer barriers than people from Macedonia and Croatia. Women reported more barriers than men. Four barriers in particular were significantly more reported by women: 'I am not in good shape', 'I do not have the energy to exercise', I am afraid of injury' and 'I do not have a safe place to exercise'

Blinde, E.M. and S.G. McCallister. *Women, disability, and sport and physical fitness activity: The intersection of gender and disability dynamics.* Research Quarterly for Exercise and Sport, 1999	USA	Qualitative, cross-sectional, interview study	16 women aged 19-54 years, with physical handicaps, cerebral parese og paraplegia, of whom 14 were wheel-chair users	Investigate the experiences of women with disabilities in sports and physical activity (focus on the role of gender and disability in the experiences)	The study found: 1) that a predominance of the movement activities in which the women participated were exercise-oriented, not sports; 2) that their primary reasons for being active were: preservation of functionality, social interaction and 'psychological factors' (e.g. reduction of stress, feeling good about themselves), 3) that they primarily experienced personal gains through activity: increased sense of physical capacity, greater self-confidence, movement as an important (fun) part of their daily lives and increased control over their life, 4) that they experienced that both their disability and their gender made it less expected and acceptable for them to be physically active (compared to non-disabled and disabled men) due to societal norms and stereotypes
Bodenmann, A., et al. *Gründe für sportliche Aktivität versus Inaktivität der Baselstädtischen Bevölkerung 1988.* Sozial- und Präventivmedizin SPM, 1990	Switzerland	Quantitative, cross-sectional, survey study	A representative sample of 778 adults, aged 16-75 years	Investigate the reasons for physical activity and inactivity in the population	The study finds that the most common reasons for physical activity were: 1) fun, well-being (27%), 2) fitness/getting in shape, functionality (20%), 3) health (16%), 4) relaxation, recreation (12%). The most common reasons for not being active were: 1) lack of time (32%), lack of interest (22%), 3) illness, injury (16%). The study found variations in the reasons in relation to 1) Age: younger people were more motivated by fun and well-being; older people were more motivated by health. Time was a greater barrier for younger people, while illness or injury was a greater barrier for older people, 2) Education: highly educated people were more motivated by relaxation, recreation, low-educated people were more motivated by health. High-educated people more often experienced lack of time and lack of interest as barriers, while low-educated people more often indicated illness, injury as a barrier
Booth, M.L., A. Bauman, and N. Owen. *Perceived barriers to physical activity among older*	Australia	Quantitative, cross-sectional, interview study	402 males and females, aged 60 years or more, divided in physically inactive vs. active	Investigate the three most common barriers to physical activity for physically active and physically inactive people, respectively	Barriers for physically inactive people: 'I am already active enough' (men 44% and women 43%). Across age, the barrier 'I am already active enough' also had the highest percentage for 60-64-year olds and 65-69-year olds, respectively (38% and 64%). For 70+ year olds, the most common barrier was having an injury

Australians. Journal of Aging and Physical Activity, 2002				Representative excerpt from the questionnaire, with questions about physical activity habits and their three primary barriers to physical activity (based on a list of 18 items)	or disability (40%). Barriers for the most physically active people: 'I am already active enough' (men and women both 60%). Conclusion: Both inactive and active people report the same barriers but in slightly different proportions
Boothby, J., M.F. Tungatt, and A.R. Townsend. *Ceasing participation in sports activity: reported reasons and their implications.* Journal of Leisure Research, 1981	United Kingdom	Qualitative, cross-sectional, interview study	254 randomly chosen citizens aged 17 or more from two socio-economically different suburban areas	Investigate reasons for ceasing participation in sports	Sports participation is strongly correlated with gender (mostly men), socioeconomic status and mobility (having a car). 43 different reasons identified in relation to ceasing participation in sports. The 6 most frequent were lack of interest, lack of facilities, poor shape/injuries, having left their association, moving, lack of time
Borodulin, K., et al. *Socio-demographic and behavioral variation in barriers to leisure-time physical activity.* Scandinavian Journal of Public Health, 2016	Finland	Quantitative, cross-sectional, survey study	2,260 physically inactive males and females aged 25-64 years	Investigate the barriers to physical activity in leisure based on a person's social background: Age, education, household income, employment status and family type	The most frequent barrier was 'lack of time'. The strongest and most systematic correlation with social background and barriers was found for: Age, occupation, status and family type. 'Lack of time' was less often reported as a barrier amongst the unemployed, single people without children and the elderly. 'Lack of motivation' was most common amongst single people without children. 'High expenses' was more frequently reported by the unemployed and less often reported in the highest income group. Conclusion: in actions to promote physical activity in leisure time, there is no single solution, as the perceived barriers vary across social background

Bragg, M.A., et al. *Motivators of and barriers to engaging in physical activity: Perspectives of low-income culturally diverse adolescents and adults.* American Journal of Health Education, 2009	USA	Qualitative, cross-sectional, interview study (focus groups)	91 young (11-15 years) and adult (18 + years), culturally diverse and of low income	Finding motives and barriers to physical activity in low-income adolescent and adult ethnic groups with low income	The motives and barriers most commonly identified amongst adolescents and adults were: social influence, time and priorities, physical environment, fun and enjoyment, intrinsic physical activities, weight concerns, fatigue, physical discomfort and current level of fitness, and immediate positive emotions. The findings were generally similar across gender, age groups, and race/ethnicity
Caperchione, C.M., et al. *Physical activity behaviours of culturally and linguistically diverse (CALD) women living in Australia: A qualitative study of socio-cultural influences.* BMC Public Health, 2011	Australia	Qualitative, cross-sectional interview study (focus groups)	110 culturally and linguistically diverse women (Bosnian, Arabic speaking, Filipino and Sudanese)	Finding motives and barriers to physical activity for ethnic women through semi-structured interviews	The most frequent themes for the interviews included: 1) knowledge of physical activity, 2) physical activity levels: all participants were more active in the country they came from because everyday life was less luxurious. E.g. they took the stairs as there was no elevator, they washed up by hand as there was no dishwasher etc. and 3) the effects of psychological and sociocultural factors - the family - prevented physical activity, 4) the environment of the surroundings was described as being unsafe and a barrier to going outside in the dark, and 5) perception of poor health and damage from physical activity behaviour - this was considered both a motivation and a barrier. Motivation because the person gets better and a barrier because the person gets hurt by physical activity. The specific themes indicated that post-war trauma, religious beliefs and obligations, socioeconomic status, social isolation, and acceptance of traditional cultural activities greatly influenced the physical activity behaviour of the ethnic women living in communities throughout Australia

Casey, M., et al. *Characteristics of physically active and inactive men from low socioeconomic communities and housing estates: A qualitative study using the socioecological model.* Annals of Leisure Research, 2011	Australia	Qualitative, cross-sectional survey and interview study	25 males, 25-65 years, of low socioeconomic status	To investigate which factors (interpersonal, organisational, societal, environmental and political influence) can influence physical activity between men with low socioeconomic status	In general, the men reported these barriers: poor health, poor economy, and that they were unfamiliar with facilities for physical activity in their local area. They had limited social support and lived in unsafe neighbourhoods
Cerin, E. and E. Leslie. *How socio-economic status contributes to participation in leisure-time physical activity.* Social Science and Medicine, 2008	Australia	Quantitative, cross-sectional, survey study	2,194 persons aged 20-65-years, healthy, English speaking	Investigate individual, socially conditioned, and environmental mediators for differences in physical activity levels between socioeconomic groups	Consistent with the ecological model, all three factors: individual, social, and environmental/surrounding factors contribute to differences in the level of activity between different socioeconomic groups. Self-efficacy and social support for physical activity explained almost all differences in physical activity regardless of education level. Physical barriers and poor access to public areas partly explained the differences between income groups in outdoor walking. (Worse outdoor areas in poorer environments). Self-efficacy and social support were key mediators of the relationship between income level and physical movement.

Cleland, C.L., et al. *Identifying solutions to increase participation in physical activity interventions within a socio-economically disadvantaged community: a qualitative study.* International Journal of Behavioral Nutrition and Physical Activity, 2014	United Kingdom	Qualitative, cross-sectional interview study	12 volunteer and paid community leaders, 8 males and 4 females, aged 25 - 65 years	Investigate benefits and barriers to the improvement of the outdoor environment in the form of construction of walking and cycling paths in a socioeconomically disadvantaged area of Belfast	Motivation factors:<br>That interventions are targeted at those whose behaviour they want to change, that those involved gain ownership of the intervention, that there is sufficient funding for the interventions, that there are local volunteers, and that interventions for physical activity are part of a longer and coherent strategy in collaboration between organisations and associations<br><br>Barriers:<br>Apathy if announced interventions are not implemented, lack of encouragement to engage in change, lack of facilities, and lack of knowledge and dissemination of knowledge about health aspects of physical activity.<br>The article concludes that the local community must target interventions for physical activity using a thorough knowledge of the target group
Cleland, V.J., et al. *Personal, social and environmental correlates of resilience to physical inactivity among women from socio-economically disadvantaged backgrounds.* Health Education Research, 2010	Australia	Quantitative, cross-sectional, survey study	291 women of low socioeconomic status mean age 48.1 years	Identify the characteristics of women from low income conditions who engage in physical activity. Characteristics are respectively divided into personal, social and environmental factors for physical activity	The strongest explanatory factors for physical activity are found on the personal level: the joy of activity and self-efficacy, whereas the social and environmental factors are weaker explanatory factors for why women with low income engage in physical activity

Costello, E., et al. *Motivators, barriers, and beliefs regarding physical activity in an older adult population.* Journal of Geriatric Physical Therapy, 2011	USA	Qualitative, cross-sectional interview study (focus groups).	31 elderlies, aged 60-94 years (mean 80 years), all with easy access to sports facilities and training staff	Investigate motivational factors for and barriers to physical activity amongst physically active and inactive elderly people	The physically active people indicated two main barriers: Time and risk of injury. As motivational factors, they stated: health (most important), socialisation, team opportunities, accessibility, facilities, fun and being encouraged by their general practitioner.<br><br>The physically inactive people indicated 6 barriers: lack of time, risk of injury, lack of self-discipline, lack of motivation, boredom and experienced intimidation (e.g. experiencing being too slow on a team).<br><br>The study concludes that tailoring intervention strategies to promote physical activity for individuals in relation to their current "Stage of Change" can result in better and more long-term behaviour change
Dyck, D.v., et al. *Who participates in running events? Socio-demographic characteristics, psychosocial factors and barriers as correlates of non-participation – a pilot study in Belgium.* International Journal of Environmental Research and Public Health, 2017	Belgium	Quantitative, cross-sectional, survey study	313 Flemish adults, aged 18-75 years, participants and non-participants in running events	1) To investigate which socioecological factors are related to participating in running events, 2) to provide an overview of the barriers to participation, and 3) to investigate differences in barriers depending on social background (gender, age and socioeconomic status)	In relation to those who did not participate in running events, the primary barriers were: poor shape, lack of time, and lack of interest. For those who participated in running events, the primary barrier was: lack of time, distance to the closest race, and economy. No differences were found in the relationship to running events across gender, age and education. This is a 'positive' trend and confirms the study's hypothesis regarding running events as having the potential to stimulate activity in population groups that are not as physically active (e.g. those with a lower education). However, this study should be interpreted with caution as the sample consisted mainly of highly educated people (79.5%)

Eakins, J.<br>*An analysis of the determinants of sports participation and time spent in different sporting contexts.*<br>Managing Sport and Leisure, 2018	Ireland	Quantitative, cross-sectional, survey study	9,346 adult persons aged 16 years or more (54 % women, 46 % men)	The purpose was to examine determinants of both sports' participation and the context in which sport is practiced. Sports participation is divided into four categories: organised training sessions, organised competition participation, occasional sports in a family context, and training alone	The study's findings concentrate on whether the socioeconomic factors vary with the context in which the activity takes place.<br>Sports participation versus those not active in sports: Those who most frequently participate in sports activities are: men, those who work part-time, the unemployed, and members of a sports club.<br>Those who least frequently participate in sports were: the elderly and people with an illness.<br>No impact on sports participation: having children, access to sports and level of education.<br>Athletes, significance of the context:<br>Women, spectators, volunteers and club members were most likely to participate in organised training. Men, volunteers, club members and spectators were more likely to participate in competitions. No consistent effect of education, labour market status and income. People with children and people who live in isolation more frequently participate in occasional sports in a family context. Men, those who live in cities, highly educated people, people with an illness participate more frequently in training alone
Emrich, E.<br>*Soziale Determinanten sportlicher Aktivitaeten im Alter – versuch einer empirischen Analyse.*<br>Sportunterricht, 1985	Germany	Quantitative, cross-sectional interview study.	41 elderly males and 34 elderly females aged 60 years or more.<br>All members of a sports association within gymnastics, athletics, swimming, rowing, table tennis and skiing.	Motivation for continued sports activity was categorised into the Continuity Hypothesis, Social Communication Hypothesis, Discrimination Hypothesis, Integration Hypothesis, and Motivation Hypothesis	There was a link between physical activity and having a good quality of life.<br>Being physically active as elderly was often associated with lifelong participation in sports, active sports within the social circle, camaraderie within the sports circle, health considerations and the joy of movement.<br>There was a gender difference in recruitment for sports participation amongst the respondents: women were from the middle and upper class, while most men were from the middle class and below
Gray, P.M., et al.<br>*Motives and barriers to physical activity among older adults of different*	United Kingdom	Qualitative, cross-sectional, interview study (focus groups)	28 elderly persons, aged 50 years or more (mean 71 years) of high and low socio-economic status	Investigate motives and barriers for physical activity amongst older adults with high and low socioeconomic status	Those with low socioeconomic status generally reported more barriers to physical activity compared to those with high socioeconomic status. Those with low socioeconomic status reported these barriers: health conditions, illness, lifestyle diseases, the outdoor conditions in the local area were considered

socioeconomic status.<br>Journal of Aging and Physical Activity, 2016					unsafe (e.g. sidewalks etc.), limited knowledge of the guidelines for physical activity as well as weather conditions.<br><br>For the group with high socioeconomic status, the barriers were: time and family, including grandchildren.<br>A motive for those with high socioeconomic status to engage in physical activity was their wish to maintain their physical activity from youth by continuing to be physically active
Grimes, P.S. and L. French.<br>*Barriers to disabled women's participation in sports.*<br>Journal of Physical Education, Recreation & Dance, 1987	USA	Quantitative, cross-sectional survey study	1,682 women with a handicap, all members of "Women Sports Foundation"	Investigate barriers to sports participation amongst women with disabilities	The main barriers to sports participation amongst women with disabilities were:<br>Lack of opportunities for physical activity/training in childhood, too few opportunities outside the framework of school, lack of interest, incompetent trainers, lack of opportunity, lack of equipment and suitable facilities.<br>Lack of role models.<br>Poor finances amongst women with disabilities
Haith-Cooper, M., et al.<br>*Exercise and physical activity in asylum seekers in Northern England; using the theoretical domains framework to identify barriers and facilitators.*<br>BMC Public Health, 2018	United Kingdom	Qualitative, cross-sectional interview study.	36 asylum seekers in Northern England from 18 countries.	Investigate barriers to physical activity for ethnic minorities in England	The barriers to physical activity were lack of: understanding of the concept of 'physical activity', knowledge of the recommendations for physical activity, and knowledge about facilities in the local area.<br>Furthermore, living as an asylum seeker due to stress and poverty as well as the temporary nature of living in an unknown place was a barrier
Hardy, S. and S. Grogan.<br>*Preventing disability through*	Great Britain	Qualitative, cross-sectional interview study (focus groups).	48 elderly persons (42 females, 6 males) aged 52-87 years	Investigate what motivates elderly people to exercise	Motives for exercising were the joy of exercising and preventing decline of health.

exercise: Investigating older adults' influences and motivations to engage in physical activity. Journal of Health Psychology, 2009					Motivation was affected by these three factors: not going out and exercising late, financial costs, and access to exercise facilities
Janssen, E., et al. *Psychosocial correlates of leisure-time walking among Australian adults of lower and higher socio-economic status.* Health Education Research, 2010	Australia	Quantitative, cross-sectional survey study	2,650 adults aged 20-65 years, of high and low socioeconomic status	Investigate psychosocial barriers for differences in physical activity between adults with different socioeconomic status	Those with low socioeconomic status reported less positive attitudes to physical activity compared with those with high socioeconomic status. Between the two groups, there were different patterns of correlations between psychosocial characteristics and leisure walking. Perceived barriers to leisure walking was strongest among those with low socioeconomic status. Therefore, interventions to promote leisure walking through the removal of perceived barriers may be more effective for those with lower socioeconomic status than for those with a higher socioeconomic status.
Kearney, J.M., et al. *Stages of change towards physical activity in a nationally representative sample in the European Union.* Public Health Nutrition, 1999	15 European countries	Quantitative, cross-sectional survey study	15,239 adults from 15 European countries	To investigate the distribution of 'stages of change' for physical activity in Europe and the influence of the sociodemographic variables on this distribution	With regard to barriers to physical activity, the barrier 'not being the sporting type' was more important to those in 'precontemplation stages', while the barrier 'work and study obligations' was more important to the people who were in the 'maintenance stage'.<br><br>There was a variation between countries. The Scandinavian countries tended to be lower in the 'precontemplation stage' and the southern countries tended to be higher (especially Greece and Portugal). Men and younger people with a higher education were more likely to be in the 'maintenance stage'

Kolland, F.<br>*Sport and physical exercise in later life.*<br>Osterreichische Zeitschrift fur Soziologie, 1992	Austria	Quantitative, cross-sectional survey study.	1000 elderly citizens aged 50 – 80+ years (mean 64.9 years).	Investigate motivational factors for remaining an active athlete in different age groups	The motives for physical activity for elderly people were generally: Health, physical ability, and social recognition<br><br>Barriers to physical activity were generally: Health problems<br><br>Continuous physical activity was most frequently seen when more than one of the motivational factors was present at one time.<br>The study also showed: that the older the age, the greater the proportion of being completely physically passive (from about 50% to about 90%. With regard to walking, hiking and swimming, there is a constant core of physically active seniors up to the age of 80, while the middle groups with moderately activity decrease with age. With regard to those who engage in a gymnastics programme (e.g. 10-20 min daily), the same increase is not seen - this group is quite constant (about 10%). The longer the schooling and the higher the social class, the more physical activity they do, and the more emphasis is placed on the health effects. Walking as a form of movement becomes more frequent with age (from 25% up to 50%)
Kondrič, M., et al.<br>*Participation motivation and student's physical activity among sport students in three countries.*<br>Journal of Sports Science and Medicine, 2013	Slovenia, Croatia and Germany	Quantitative, cross-sectional survey study	390 sports students, mean age 22.4 years	Investigate young people's motives for engaging in physical activity	The six most prominent motives for engaging in physical activity were: engaging in physical activity with a friend, popularity, fitness and health, social status, participation in sporting events and relaxation through the activities.<br>The difference between genders (which also applied to all three countries): men's motivation for physical activity was to gain popularity in society and amongst friends, where women's motives were that they experience movement as a means of relaxation, which in principle strongly correlates with male and female stereotypes and roles

Lattimore, D., et al.<br>*Self-reported barriers of middle-aged and older adults entering a home-based physical activity program.*<br>Californian Journal of Health Promotion, 2011	USA	Mixed methods study, pre and post intervention survey and interview	380 healthy, physically inactive citizens from 3 American States aged 50 years or more	Find barriers to a telephone-guided home exercise programme.<br>Sub-study in "Active for life". The intervention was: a face-to-face counselling session, followed by telephone counselling.<br>BMI over 30 was compared to BMI under 30.<br>African Americans were compared to whites.	Overall, the barriers fell into three main categories:<br>1) Personal: Health (most prevalent amongst women and the elderly) and lack of motivation (most prevalent amongst men).<br>Lack of motivation as a factor decreased with age.<br>Racial difference in health as a limiting factor: Significantly fewer African Americans used health as a limiting factor.<br>2) Environment: (time constraints and the weather)<br>Time constraints as a limitation decreased with age.<br>Cold and wet weather was a barrier to getting outside.<br>3) Social barriers: Very few considered it a barrier (below 5%), no association with either gender or race.<br><br>Overall, the study shows that there are unique barriers within different groups of elderly people and that individual approaches to motivation and activity can be effective.
Lee, C. and Brown, W.J.<br>*Australian migrant women and physical activity : attitudes, barriers, preferences and participation.*<br>ACHPER Healthy Lifestyles Journal, 1998	Australia	Quantitative, cross-sectional, survey study.	202 female immigrants and second-generation immigrants from Europe (Macedonia, Greece, Netherlands, Poland) mean age 45.2 years.	To investigate barriers and motives for physical activity in female immigrants	Most frequently mentioned motivations for physical activity: A group to exercise with, exercise videos, and motivation from a general practitioner/healthcare professional.<br>Most frequent barriers to physical activity:<br>Lack of a group to exercise with (which is a more important factor than for Australians), laziness, and lack of time.<br><br>There was no difference in preferences between the different stages of change, both in terms of motivations and barriers. But there was a slight difference in which sports disciplines were preferred (which reflected the culture of the home country).<br>Immigrants from non-English speaking countries had a greater proportion of completely physically inactive people than Australians.

Lehr, U. *Gymnastics and sport in old age.* Aktuelle Gerontologie, 1981	Germany	Literature study	Elderly in general	Review of motivational factors and barriers in relation to physical activity in the elderly.	In motivating the elderly, it is important to motivate by emphasising the personal "benefit" the activity can provide. This, as a whole, conveys the instrumental motivation ("it is healthy") with the expressive motivation ("it is fun").<br>Barriers to physical activity in the elderly: negative self-esteem, previous lack of sports activity, physical disabilities, poor social experiences with sports (e.g. competitions), fear of social contact, and environmental factors, such as water splashing, slippery floors and noisy children in the swimming pool.<br>The author advocates that offers for the elderly must be differentiated in relation to the individual target group
Lusmägi, P., et al. *Changes in leisure-time physical activity levels and perceived barriers among Estonian adults over a two-year period.* Acta Kinesiologiae Universitatis Tartuensis, 2018	Estonia	Quantitative, cross-sectional survey study. (Two cross-sectional waves (2013 and 2015) of a national running survey).	1,009 Estonian citizens in 2013, 1,004 in 2015	1. Describe changes in LTPA (Leisure Time Physical Activity) levels amongst Estonian adults over a two-year period.<br>2. Investigate the relationship between LTPA levels, socio-economic status and health indicators.<br>3. Investigate perceived barriers to physical activity in relation to gender, age, and level of education.	The main self-assessed barriers: Lack of interest, not wanting to exert oneself and fatigue after work. In addition, for the 50-74-year-olds: Poor health. Mentioned to a lesser degree was: Lack of access to sports facilities and the price.<br>Perceived barriers varied with level of education: Those with a low level of education placed more emphasis on lack of access to sports facilities and poor health than those with a high level of education.<br>In general, the level of physical activity in the adult population were quite low.<br>Significant predictors of physical inactivity: old age, low level of education, and dissatisfaction with one's own health
Martins, J., et al. *Adolescents' perspectives on the barriers and facilitators of physical activity: A systematic review*	No restrictions on country	Systematic literature review of qualitative studies	Adolescents aged 13-18 years	To investigate barriers and facilitators for physical activity	The main facilitators for physical activity: Attitude towards physical activity, the person's motivation, the person's perception of competencies and body image, that it is fun, good physical environment, and easy access.<br>Only specific life transition periods were referred to as a barrier

of qualitative studies. Health Education Research, 2014					
McGuire, A., C. Seib, and D. Anderson. *Factors predicting barriers to exercise in midlife Australian women.* Maturitas, 2016	Australia	Quantitative, cross-sectional, survey study.	225 voluntary female participants aged 40-65 years	To investigate perceived barriers to exercise	The perception of barriers to exercise correlates with the belief in health benefits of exercise, exercise self-efficacy as well as physical and mental well-being. Together, these factors explained 41% of the variation in perceived barriers to exercise. These variables were negatively correlated with the perception of barriers, which means that the lower self-efficacy and lower physical and mental well-being a person has, the higher the perception of barriers to physical activity
Nau, T., G. Nolan, and B.J. Smith. *Promoting adherence to organised physical activity among socially disadvantaged older people.* Ageing and Society, 2019	Australia	Qualitative, cross-sectional interview study (focus groups)	42 socially disadvantaged elderly people (mean age 76 years)	To investigate promoting factors and adherence to physical activity (rehabilitation courses etc.) for socially disadvantaged elderly persons	Barriers to physical activity for socially disadvantaged elderly persons were: deteriorating health, lack of belonging, and lack of motivation.<br><br>The study concludes that belonging can be promoted by creating positive and inclusive experiences, ensuring stigma-free environments, and providing opportunities to create personal connections. Motivation can be improved by ensuring that activities are of interest and functional benefit, rehabilitation programmes are appropriate and appealing, and enjoyment is prioritised.

Osuji, T., Lovegreen, S., Elliott, M., Brownson,R.C. *Barriers to physical activity among women in the rural midwest.* Women & Health, 2006	USA	Quantitative, cross-sectional survey study (telephone survey)	2,510 women aged 18-94 years (mean 48), from rural Southeastern Missouri, Tennessee, and Arkansas, living less than 2 miles from a public walking trail	Investigate the influence of gender and income groups on the level of physical activity and the self-perceived personal and environmental barriers to physical activity.<br>The dependent variable is the probability of not meeting the recommendations for physical activity (Moderate physical activity for at least 150 minutes per week)	The most common barriers to physical activity reported by more than 50% of the participants: fatigue, lack of time, bad weather, lack of energy and lack of motivation.<br>Finds dose-response relationship between the number of barriers to physical activity and the probability of not meeting the recommendations for physical activity. The dose-response relationship was most pronounced in women in high-income groups. The reason is unclear.
Palmer, T. and C.A. Jaworski. *Exercise prescription for underprivileged minorities.* Current Sports Medicine Reports, 2004	USA	Literature review	Women of ethnic minorities	Literature review of studies regarding the socio-economic groups participating in least physical activity: women and ethnic subgroups	Self-efficacy seems to be the most important potential factor for motivating populations to increase physical activity, as increased self-efficacy will cause them to view obstacles as challenges. Self-efficacy is boosted by physical activity, so a main goal is to get people to start with physical activity. Walking is recommended as it is cheap, safe, and can easily be implemented on a weekday.<br><br>Perception of barriers to physical activity varies between population groups, with differences in socioeconomic status as the main reason for differences in activity level.<br>Both physically active and inactive people indicated the same barriers: lack of willpower, motivation and energy, health problems, which may mean that clearing barriers out of the way does not necessarily improve physical activity.<br><br>The study concludes that tailored interventions have a better effect on people's physical activity.

Pan, S.Y., et al. *Individual, social, environmental, and physical environmental correlates with physical activity among Canadians: A cross-sectional study.* BMC Public Health, 2009	Canada	Quantitative, cross-sectional survey study	5,167 Canadians aged 15–79 years.	To investigate barriers and motivational factors for physical activity across different social backgrounds	The study shows that family income, self-rated health, intention, self-efficacy, perception of barriers, and availability of facilities were associated with physical activity.<br>The effects of the perceived health benefits, education and family income were more salient to older people, while the influence of education was more important for women, and the influence of the barriers experienced was more prominent for women and younger people.<br>Availability of facilities was more strongly associated with physical activity amongst people with a university degree than amongst people with a lower level of education.
Pano, G. and L. Markola. *14-18 years old children attitudes, perception and motivation towards extra curricular physical activity and sport.* Journal of Human Sport and Exercise, 2012	Albania	Quantitative, cross-sectional survey study.	1,062 high-school students aged 14 to 18 years (465 males and 597 females)	To investigate high school students' motives and barriers to physical activity and sports	The motives for physical movement: it should be fun and improve their physical image.<br><br>The barriers to physical activity: lack of recreational and sports facilities. The study concludes that it is thus important to offer more sports facilities and opportunities for this target group in order to increase their physical activity
Pedersen, P.V., et al. *Readiness to change level of physical activity in leisure time among physically inactive Danish adults.* Scandinavian Journal of Public Health, 2009	Denmark	Quantitative cross-sectional, survey study	9,160 physically inactive Danish adults aged 16-79 years	To investigate 'readiness to change' in the level of physical activity in leisure time amongst physically inactive adults and across sociodemographic variables	52% of the physically inactive participants were ready to change their level of physical activity.<br>Men had greater odds of being ready to change their behaviour than women. Readiness to change decreased with age and increased with the person's level of education. Those who were ready to change behaviour led an active and social lifestyle that was characterised by a significant health-oriented commitment, while the opposite characterised those who were not ready to change behaviour. Those who were ready to change their behaviour wanted help and support from their family. Those who were not

					ready to change behaviour wanted help from a general practitioner or they did not want help at all
Pelssers, J., et al. *Acting one's age in physical exercise: do perceived age norms explain autonomous motivation among older adults?* Journal of Aging and Physical Activity, 2018	Belgium	Quantitative cross-sectional survey study	409 elderly persons aged 55 or more (mean age 68.7 years, 54 % men)	Investigate whether perceived exercise norms are associated with higher levels of motivation	Older adults who perceived more positive exercise norms were more autonomously motivated to exercise. The findings indicate that social identity approach and self-determination theory can be meaningfully integrated
Perracini, M.R., et al. *Physical activity in older people – Case studies of how to make change happen.* Best Practice and Research: Clinical Rheumatology, 2017	Countrys not mentioned	Qualitative literature review	Elderly people aged 50 years or more.	The purpose is to update the recommendations for physical activity for the elderly. In this context, a number of motivational factors and barriers to physical activity among the elderly are outlined on the basis of a systematic review (Sun et al. 2013)	Elderly people's motivation for participation in physical activity is: interaction with peers, joy in encouragement from others, joy in professional instruction, achieving better physical capacity, better self-confidence, physical independence, improved health and mental well-being. Barriers are: experience of social awkwardness in connection with physical activity with others, physical limitations (pain or discomfort, worry about falling, worry about co-morbidities), difficulty accessing activities (access to activities, price of activities), prioritisation of non-sports related activities, negative beliefs (apathy, belief that sports are irrelevant and inefficient, the power of habit)

Poobalan, A.S., et al.<br>*Physical activity attitudes, intentions and behaviour among 18-25 year olds: A mixed method study.*<br>BMC Public Health, 2012	Scotland	Mixed methods, survey and focus group interview study.	1,013 young people aged 18-25 years participated in a survey, of whom 26 participated in focus group interviews.	To investigate physical activity in young people and the factors that can influence it, including attitudes, motives and barriers	Joy, appearance and feeling comfortable were important motives for this group to engage in physical activity. The study concludes that a targeted intervention in relation to improving and maintaining physical activity for this target group must incorporate these motives for activity
Rauzon, T.A.<br>*Barriers to participation in physical activity/exercise for women with physical disabilities*.<br>2002.<br>ProQuest Information and Learning Company	USA	Quantitative cross-sectional survey study	149 women aged 18-64 years, all with a physical handicap	1) What barriers restrict women with physical disabilities from physical activity?<br>2) Are the limitations comparable to the limitations experienced by women without disabilities?<br>3) Is there a relationship between activity behaviour (exercise behaviour) and the onset of disability?	1) Social support from family and friends; belief in being able to engage in physical activity; barriers in the environment such as lack of time, price, access to facilities, and transport options were barriers to physical activity.<br>2, 3) No differences could be detected due to the low number of participants.<br><br>The study advocates a greater focus on appealing more to women with disabilities so that it is easier for them to envision themselves engaging in physical activity
Recours, R.A., M. Souville, and J. Griffet.<br>*Expressed motives for informal and club/association-based sports participation.*<br>Journal of Leisure Research, 2004	France	Quantitative cross-sectional survey study.	871 secondary school students aged 14-19 years (432 men, 439 women)	To investigate the motives for informal association-based sports participation	The results show that women primarily were motivated to sports due to the social element, whereas men to a higher extent were driven by the competitive element.<br><br>Across the groups, intrinsic motivation was more important for physical activity than extrinsic motivation

Romeike, K., et al.<br>*Similarities and differences in underlying beliefs of socio-cognitive factors related to diet and physical activity in lower-educated Dutch, Turkish, and Moroccan adults in the Netherlands: a focus group study.*<br>BMC Public Health, 2016	Netherlands	Qualitative, interview study (semi-structured focus groups)	90 lower-educated Dutch, Turkish, and Moroccan men and women aged 22 – 73 years	Investigate barriers related to physical activity and healthy diet in people with low education from the Netherlands, Turkey and Morocco	Social support can motivate towards an increased level of physical activity. Physical activity together with others was a motivating factor.<br><br>Barriers to physical activity were: Lack of time and fatigue. There were barriers due to religion and culture - men and women might not exercise in the same place. This was perceived as a barrier amongst Moroccans and Turks (not a factor for the Dutch)
Santos, I., et al.<br>*Motivation and barriers for leisure-time physical activity in socioeconomically disadvantaged women.*<br>PLoS ONE, 2016	Australia	Quantitative, 3-year follow-up survey study	1,664 socio-economically disadvantaged women aged 18-46 years	To investigate the cross-sectional and longitudinal relationship between motivation and barriers to physical activity and physical activity behaviour in women who are financially disadvantaged. The study also examined whether intentions for weight control moderate these relationships	Intrinsic motivation was associated with physical activity in leisure time by women who are financially disadvantaged. The findings also show that women who attempt to lose or maintain weight loss report higher levels of intrinsic motivation. I.e. internal motivation for physical activity is a significant factor in maintaining one's physical activity over time for women trying to control their weight.

Shifflett, B., C. Cator, C., and Megginson, N. *Active lifestyle adherence among individuals with and without disabilities.* Adapted Physical Activity Quarterly, 1994	USA	Quantitative, cross-sectional survey study.	203 bachelor students, 141 without, 62 with physical disabilities. (38% men and 62% women, mean age 26.6 years)	To investigate adherence to an active lifestyle for individuals with and without physical disabilities	Barriers to adherence to physical activity for those without a disability: lack of time, lack of motivation, having active friends, and lack of facilities in the area.<br>Barriers to adherence to physical activity for those with one or more disabilities: lack of time and physical limitations due to the disability.<br>The mean values of barriers to physical activity were higher for people with a disability than without a disability, suggesting that these persons find health issues and access to facilities (which were the barriers the study addressed) as greater barriers to maintaining an active lifestyle than those without a disability.<br>In contrast, the mean values for a majority of social support items (as a barrier to physical movement) were lower for people with a disability, indicating that the lack of active friends to do things with may be less important to this group than injuries or health problems
Stappen, V.v., et al. *Barriers from multiple perspectives towards physical activity, sedentary behaviour, physical activity and dietary habits when living in low socio-economic areas in Europe. The Feel4Diabetes Study.* International Journal of Environmental Research and	Six European countries (Belgium, Bulgaria, Finland, Hungary, Greece, Spain)	Qualitative, cross-sectional focus group interview study.<br><br>3 focus group interviews in each country, one with parents, one with teachers, and one with local community workers	115 parents of primary schoolchildren (6–12 years old) living in vulnerable areas (86.5 % female parents)	To investigate the barriers to physical activity that young families from vulnerable areas experience, sedentary behaviours and dietary habits (three important lifestyle behaviours for the prevention of type-2 diabetes)	This study identified some general barriers for people with low socioeconomic status at all levels of the socioecological model for health behaviour: the individual level (lack of energy and motivation for physical activity); interpersonal level (lack of time for physical activity and financial constraints); organisational level (lack of facilities in the neighbourhood and at home for physical activity) and macro/public policy level (adverse weather conditions was a barrier to physical activity).<br>Additional barriers were: lack of parents' knowledge and lack of parents' skills, being provided for by other stakeholders (i.e. teachers and community workers).<br>The results of this study demonstrate the added value of including multiple perspectives when developing a lifestyle intervention aimed at preventing type 2 diabetes in vulnerable groups. Future lifestyle interventions are recommended to include several components (family, school and community) and can

Public Health, 2018					be implemented across European countries if country-specific adaptations are allowed
Stavridis, A., S. Kaprinis, and I. Tsirogiannis. *Participation's motives in dancing activities. Gender and age as differentiation factors.* Mediterranean Journal of Social Sciences, 2015	Greece	Quantitative, cross-sectional survey study	220 Performance dancers aged 15 to more than 60 years	Investigate what motivates people to participate in dance activities, including special performance dance and see if there is a difference with regard to gender and age	The motive that resonates the most is the dancers' intrinsic motivation, that they enjoy the nature of the activity and that they find joy in the activity. Across gender, there is no difference in the motives for dancing. In terms of age, younger people have more intrinsic motivation than older people
Stødle, I.V., et al. *The experience of motivation and adherence to group-based exercise of Norwegians aged 80 and more: A qualitative study.* Archives of Public Health, 2019	Norway	Qualitative study, cross-sectional, interview study (semi-structured interview)	3 men and 4 women aged 81-92 years	To describe older people's motivation to participate in exercise and why they are persistent in a group-based exercise intervention in their local area	The participants found it motivating to participate, as they were motivated by social and professional support (family, general practitioner, etc.), it increased the elderly persons' motivation for physical activity.<br>Participation in the group's exercise sessions meant positive changes in their physical, mental, and social functions that improved their motivation to maintain their participation and it led to positive behavioural changes that were important for their daily lives

Taylor, T. and K. Toohey. *Ethnic barriers to sports participation.* Australian Parks & Recreation, 1995	Australia	Literature review	Non-European immigrants to Australia	To investigate barriers to sports participation in ethnic groups	Barriers to physical activity may be religiously imposed by clothing and patriarchal patterns that prohibit women from choosing, rather than the barriers having anything to do with the sport itself. Non-European immigrants (especially women) were severely under-represented in sports participation. Some immigrants use sports participation as a means of maintaining their ethnic/cultural identity, but sports were also used for acculturation in the new country
Tischer, U., I. Hartmann-Tews, and C. Combrink. *Sport participation of the elderly – the role of gender, age, and social class.* European Review of Aging and Physical Activity, 2011	Germany	Literature overview of results of cross-sectional and longitudinal data sets concerning sport participation of the elderly	Elderly citizens	To investigate what motivates older people to sport participation	A healthy lifestyle was the most discussed motivating parameter in adults and the elderly. The parameters 'fun and enjoyment' were far less prominent for the elderly than for the young adults
Úbeda-Colomer, J., J. Devís-Devís, and C.H.P. Sit. *Barriers to physical activity in university students with disabilities: Differences by sociodemographic variables.* Disability and Health Journal, 2019	Spain	Quantitative, cross-sectional survey study.	1,219 university students with a handicap (592 men, 622 women, 5 missing) aged 18 to 76 years old.	To identify the barriers to physical activity among university students with a disability and between different sociodemographic variables	Based on the socioecological model, the main barriers were personal: fatigue, pain, lack of motivation. This was especially the case for women, students with multiple disabilities, those with a higher degree of disability and the elderly, followed by the organisational barriers (lack of tailored programmes and financial costs), which was especially true for those with multiple disabilities, a physical disability and those with a higher degree of disability.<br>At the interpersonal level, inactivity with one's friends and family were the biggest barriers to physical activity, this was especially true for those with multiple disabilities and the elderly.<br>Holes in the streets or other public spaces were the biggest barrier at the community level, this was especially true for those with multiple, physical, or a higher degree of disabilities.

					The groups that had the most barriers to physical activity were generally those with multiple disabilities and with a higher degree of disability. The results of the study show that there is a need for interventions to promote physical activity that address the key barriers that exist at each level of the socioecological model.
Varsamis, P. and A. Papadopoulos. *Effects of disability and gender on the perceived reasons for participating in sport.* International Journal of Learning, 2013	Greece	Mixed methods, cross-sectional study. (guided interview, cognitive tests by psychologist, motor functions' assessments by physical education teachers)	19 males and 19 females, mean age 20 years, with physical og mental disabilitie.	Investigate motives for participation in physical activity amongst young people with physical/mental disabilities	Overall, the motivation was similar to that found in the normal population. The 5 most frequently stated motivational factors are: Health and wellness, social interaction and friendship, fun and entertainment, effect of physical exercise, and getting to know the limits and performance of one's body
Verloigne, M., et al. *Mediating effects of self-efficacy, benefits and barriers on the association between peer and parental factors and physical activity among adolescent girls with a lower educational level.* PLoS ONE, 2016	Belgium	Quantitative, cross-sectional survey study	226 female teenagers mean age 16 years, pupils on secondary schools.	Investigate the importance of friends and parents for young girls' physical activity habits as well as investigate mediating personal factors such as self-efficacy, perceived benefits, and barriers	The study found positive correlations between friends and parents' 1) frequency of participation in physical activity, 2) frequency of engaging in physical activity with the respondent and 3) frequency of invitations to the respondent to engage in physical activity and the respondent's physical activity level (sports and active transport). The mediating personal factors were consistent: 1) self-efficacy in relation to physical activity, 2) the joy of being active, 3) that physical activity counteracts boredom, and 4) socialising with friends and meeting new people (the latter, however, was only mediating between friend factors and the respondent's physical activity level). In turn, the study found that perceived barriers to physical activity (not being good at physical activity or experiencing a lack of transportation options to facilities) mediated the same conditions. The only barrier that mediated select contexts was not liking engaging in physical activity.

White, S.M., Wójcicki, T.R. and McAuley, E. *Social cognitive influences on physical activity behavior in middle-aged and older adults.* Journals of Gerontology – Series B Psychological Sciences and Social Sciences, 2012	USA	Quantitative, longitudinal survey study, with an 18-month follow-up period	$N_{baseline}$=321<br>$N_{follow-up}$=227<br>middle-aged and elderly university employees, mean age 63.8 years, 80% women	Investigate the applicability of 'Social Cognitive Theory' (Bandura) to explain the physical activity pattern of middle-aged and older people (both direct and indirect effects)	The study identified direct and indirect positive relationships between self-efficacy (operationalised as their belief in being able to maintain regular physical activity) and the level of physical activity (scale with ten items, PASE). Self-efficacy thus also had a significant positive effect on the respondents' expected physical benefits from physical activity, which affected the level of physical activity.<br>The study also found that self-efficacy was positively correlated with expected physical effects, self-image, and social gains from physical activity as well as with set goals for physical activity behaviour and the experience of fewer physical limitations for physical activity. Finally, changes in self-efficacy from baseline to follow-up were correlated with changes in the same variables as described above.
Young, D.R. and King, A.C. *Exercise adherence: determinants of physical activity and applications of health behavior change theories.* Medicine, Exercise, Nutrition & Health, 1995	USA	Literature review	Older individuals with obstacles against engagement in physical exercise: smokers, the overweight, those with low self-efficacy for exercise, and other groups	Review of determinants of physical activity behaviour, categorised into<br>1) personal characteristics,<br>2) environment,<br>3) the physical intervention itself	The motivational factors: belief in health effects and belief in being able to be physically active (exercise self-efficacy).<br>Barriers: Lack of time, lack of motivation, fear that sports are dangerous (especially in the elderly)<br>1) demographic factors, health status, knowledge and confidence in health effects as well as previous experience with physical activity were discussed.<br>It is recommended to focus on the factors that are influential when changing people's motivation for physical activity. For example, knowledge, attitudes, self-efficacy, and perception.<br>2) social support, life events, nudging, accessibility to sports facilities were discussed. Attention to risk factors for dropout and instruction in dropout prevention are pointed out as important.<br>3) There should be a focus on effective ways to remove barriers, as well as developing activities with a broad appeal (e.g. walking for the elderly and women)

Zunft, H.J.F., et al. *Perceived benefits and barriers to physical activity in a nationally representative sample in the European Union.* Public Health Nutrition, 1999	15 European Union countrys	Quantitative, cross-sectional survey study, using an interview-assisted face-to-face survey	15,239 EU citizens, classified according to sex, age (15-34, 35-54, 55+) and highest level of education attained (primary, secondary, or tertiary)	Investigate people's perceptions of motives and barriers to physical activity between different social backgrounds (age, gender, education)	The most important motivation for physical activity was to maintain good health, to 'loosen up' the body, and to get in good shape.<br>The importance of the health argument was highest amongst the elderly and for people with a basic education.<br>The most important barrier to increasing physical activity was work or study obligations. The next barrier is not being the 'sporty type'. There are different patterns between the countries. In Denmark, 35% found it motivating to engage in physical activity to socialise with others, while in the rest of the countries the percentage was 6-22%
